# Biallelic truncation variants in *ATP9A* are associated with a novel autosomal recessive neurodevelopmental disorder

**DOI:** 10.1038/s41525-021-00255-z

**Published:** 2021-11-11

**Authors:** Francesca Mattioli, Hossein Darvish, Sohail Aziz Paracha, Abbas Tafakhori, Saghar Ghasemi Firouzabadi, Marjan Chapi, Hafiz Muhammad Azhar Baig, Alexandre Reymond, Stylianos E. Antonarakis, Muhammad Ansar

**Affiliations:** 1 Center for Integrative Genomics, University of Lausanne, Lausanne, Switzerland; 2 Neuroscience Research Center, Faculty of Medicine, Golestan University of Medical Sciences, Gorgan, Iran; 3 Anatomy Department, Khyber Medical University Institute of Medical Sciences (KIMS), Kohat, Pakistan; 4 Iranian Center of Neurological Research, Neuroscience Institute, Tehran University of Medical Sciences, Tehran, Iran; 5 Genetics Research Center, University of Social Welfare and Rehabilitation Sciences, Tehran, Iran; 6 Department of Biotechnology, Institute of Biochemistry, Biotechnology and Bioinformatics, The Islamia University of Bahawalpur, Bahawalpur, Pakistan; 7 Department of Genetic Medicine and Development, University of Geneva Medical Faculty, Geneva, 1211 Switzerland; 8 Medigenome, Swiss Institute of Genomic Medicine, Geneva, Switzerland; 9 Present Address: Jules-Gonin Eye Hospital, Department of Ophthalmology, University of Lausanne, 1004 Lausanne, Switzerland; 10 Institute of Molecular and Clinical Ophthalmology Basel (IOB), Basel, Switzerland

**Keywords:** Molecular medicine, Consanguinity, Disease genetics

## Abstract

Intellectual disability (ID) is a highly heterogeneous disorder with hundreds of associated genes. Despite progress in the identification of the genetic causes of ID following the introduction of high-throughput sequencing, about half of affected individuals still remain without a molecular diagnosis. Consanguineous families with affected individuals provide a unique opportunity to identify novel recessive causative genes. In this report, we describe a novel autosomal recessive neurodevelopmental disorder. We identified two consanguineous families with homozygous variants predicted to alter the splicing of *ATP9A* which encodes a transmembrane lipid flippase of the class II P4-ATPases. The three individuals homozygous for these putatively truncating variants presented with severe ID, motor and speech impairment, and behavioral anomalies. Consistent with a causative role of *ATP9A* in these patients, a previously described *Atp9a−/−* mouse model showed behavioral changes.

## Introduction

Intellectual disability (ID) or delayed psychomotor development are common and highly heterogeneous phenotypes of genetic origin, affecting 1–3% of the general population^1,2^ which pose a significant socio-economic burden on the affected families, the health care system, and society in general^3^. Despite considerable progress in genetic diagnosis after the introduction of high throughput sequencing technologies, the genetic cause of more than half of ID cases remains undetermined^4^. The leading genetic cause of ID in individuals from outbred populations is de novo variants^5,6^; in contrast a substantial fraction of autosomal recessive (AR) disorders cause ID in families with multiple affected individuals that practice consanguinity^7^. It is estimated that worldwide 10.4% of marriages occur among close relatives^8^. Consanguinity increases the extent of homozygous genomic regions and brings to homozygosity deleterious alleles resulting in birth defects and infant mortality^9,10^. Large consanguineous families with (multiple) affected individuals thus provide a unique opportunity to identify novel recessive causative genes.

P4-ATPases are transmembrane lipid flippases^11^, that function in vesicles formation and trafficking. They regulate the asymmetric distribution of phospholipids in membranes of eukaryotic cells^11,12^. There are 14 different P4-ATPases in humans that can be phylogenetically grouped in five classes^13^. ATP9A and its 75% similar paralog ATP9B are the unique members of class II. They are the only P4-ATPase that do not require the CDC50 β-subunit for normal function and cellular localization^14^. They show different intracellular and tissue distribution: ATP9A is found in early and recycling endosomes and at a lower level at the plasma membrane, while ATP9B is only found in the trans-Golgi network^12,14–16^. Similarly, the genes encoding ATP9A and ATP9B present with overlapping but different expression patterns with *ATP9A* mainly expressed in the brain (Human Protein Atlas, GTEx). Suggestive of an important role of ATP9A in intercellular communication, this P4-ATPase inhibits extracellular vesicles release^15,16^.

Here we report two consanguineous families with homozygous pathogenic variants predicted to alter *ATP9A* splicing and we propose ATP9A as a novel cause of a recessive neurodevelopmental disorder.

## Results

### Clinical report

**Fig. 1:**
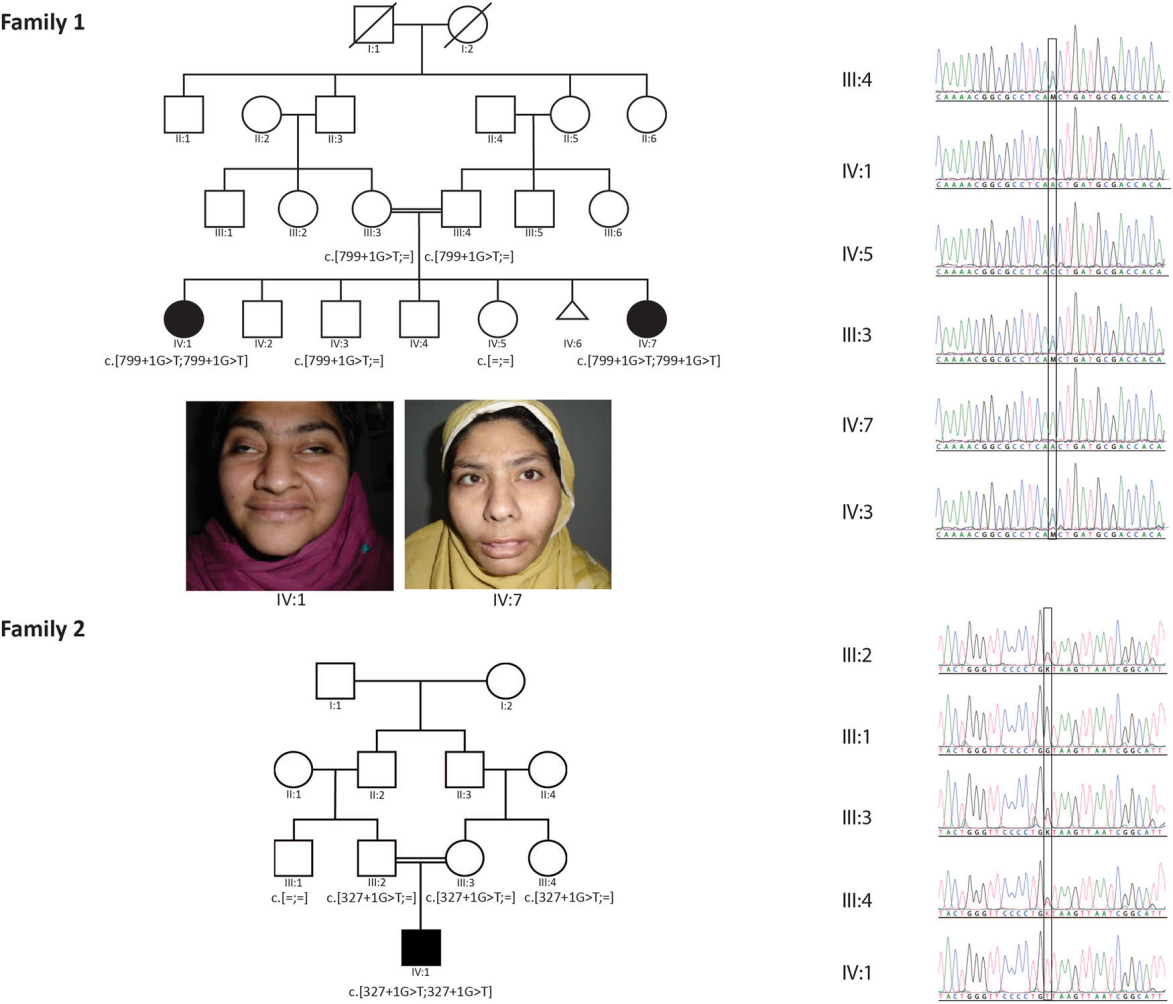
Pedigrees and Sanger sequencing. The pedigrees and the available genotypes of the Pakistani (family 1, top) and Iranian families (family 2, bottom) are depicted on the left. Sanger sequencing chromatograms confirming the segregation of the *ATP9A* NM_006045.3:c.799 + 1 G > T (six top traces) and NM_006045.3:c.327 + 1 G > T variants (bottom five traces) are shown on the right. Inserts showing the facial features of the two affected sisters IV:1 and IV:7 of family 1 are presented below the Pakistani pedigree.

We identified three affected individuals from two unrelated consanguineous families of Pakistani and Iranian origin. The main clinical features of the affected individuals are reported in Table 1 and in Fig. 1.

**Table 1:** Clinical features of patients with homozygous *ATP9A* splicing variants.

Family	1	1	2
Individual	IV:1	IV:7	IV:1
Sex	F	F	M
Origin	Pakistani	Pakistani	Iranian
Consanguineous parents	Yes	Yes	Yes
Age at last evaluation (years)	28	21	11
ATP9A variant (gDNA)	Chr20:50305602 C > A	Chr20:50305602 C > A	Chr20:50342357 C > A
General characteristic			
Head circumference (cm)	51	54	53
Height (cm)	149	169	140
Weight (kg)	64	67	45
Microcephaly	+	−	−
Strabismus	+	+	−
Facial dysmorphism	+	+	+
Neurodevelopment			
Severe Intellectual disability	+	+	+
Motor delay	+	+	+
Speech delay/ dysfunction	+	+	+
Fine motor impairment	+	+	+
Epilepsy	–	–	+
Brain MRI anomalies	n.d.	n.d.	–
Behavioral anomalies			
ADHD	+	+	n.d.
Stereotypic movement	n.d.	n.d.	+
Autistic features	–	–	+
Aggressiveness	+	+	n.d.

*n.d.* not determined, *ADHD* attention deficit hyperactivity disorder.

Family 1 is from the Khyber Pakhtunkhwa region of Pakistan. As indicated in the pedigree, the unaffected parents (III:3 and III:4), who are first cousins, have six children. The oldest and youngest siblings (IV:1 and IV:7) exhibited similar clinical features that include delayed childhood milestones, severe ID, mild hypotonia, attention deficit hyperactivity disorder (ADHD), aggressive behavior, bilateral eye squints, and impaired vision. The oldest affected daughter (IV:1) presented with microcephaly (<1st percentile, −3.12 SD), however the head circumference of the second affected sibling, the youngest daughter, (IV:2) is in the normal range (39th percentile). We could not perform brain magnetic resonance imaging (MRI) because the family lives in a very remote area and did not agree to travel due to COVID19 outbreak and the high rate of infections in the region. While the other siblings (IV:2, IV:3, IV:4, and IV:5) were unaffected, we note that pregnancy IV:6 was not carried to term (Fig. 1).

The proband (IV:1) of the Iranian family 2 is the only child born from a couple of first cousins (Fig. 1). Childbirth was unremarkable. The parents noticed a delay in the development of both language and walking (18 months). The proband started epileptic episodes at 3 years of age and seizures were controlled with sodium valproate. An abnormal EEG with epileptiform activity was reported. Brain MRI was normal. At the time of the last visit, the child did not present motor paralysis or coordination deficit, but he had an abnormal gait. At 11 years of age, height, weight, and head circumference were in the normal range with 140 cm, 45 kg, and 53 cm, respectively. Eye contact was impaired and there was complete language dysfunction. He is presenting with severe ID, prominent stereotypic movement disorder, and autistic features. The proband has arched eyebrows with round, downturned eyes, thin lips, bulbous nose, and a short philtrum. The proband’s cousin was also reported to be affected by a neurodevelopmental disorder. He is presenting with moderate ID, autistic features, and epilepsy. However, he does not have any motor or coordination problem. The different severity of ID, growth parameters, and the absence of motor impairment are possibly indicative of a different genetic etiology.

### Exome analysis

In family 1, whole-exome sequencing (WES) was performed in the proband (IV:1) to exclude variants in genes previously reported to cause ID or developmental delay. Subsequently, SNP-array was performed in both affected individuals (IV:1 and IV:7), parents (III:3 and III:4) and an unaffected sibling (IV:3). Homozygosity mapping revealed a 2.5 Mb region of homozygosity (chr20[GRCh37]:49010965-51638043) common in both patients (IV:1 and IV:7) but not in the parents (III:3 and III:4) and an unaffected sibling (IV:3). In total, six homozygous variants from the WES data of the proband (IV:1) were present in the segregating ROH (chr20:49010965-51638043) (as mentioned in the Supplementary Table 1), but the splicing variant (NM_006045.3:c.799 + 1 G > T) in *ATP9A* was the only mutation with the MAF < 1% (in any of the population in the gnomAD database). (Fig. 1). The variant was not present in gnomAD^17^, Bravo (https://bravo.sph.umich.edu/freeze5/hg38/) or our local database of >500 Pakistani controls. Its segregation in the family was confirmed by Sanger sequencing, in particular, the youngest sister and second affected sibling is homozygous for this variant (Fig. 1). The change at the conserved first nucleotide of the donor splice site was predicted to cause abnormal splicing by SpliceAI^17^ (score DS_DL = 0.99), MaxEntScan^18^ (MaxEntScan_diff = 8.504), and NNsplice^19^. RNA samples from affected individuals were not available to assess RNA splicing.

Our search for more cases led to the identification of a second family. The WES of proband IV:1 from family 2 also revealed the presence of a homozygous splicing variant in *ATP9A*, a base pair substitution in intron 3 of *ATP9A* (NM_006045.3:c.327 + 1 G > T). This variant is absent from the gnomAD^17^ and Bravo databases, the Iranome (i.e. 800 healthy individuals from eight different Iranian ethnic groups, http://www.iranome.ir/) and our local database of >250 Iranian controls. Multiple predictions tools indicated a likely loss of the canonical donor splice site (NNsplice, SpliceAI score DS_DL = 0.95, MaxEntScan_diff = 8.504). The abnormal splicing could either result in the skipping of the inframe exon 3, leading to the deletion of 38 amino acid residues, or utilization of an alternative donor site resulting in partial intronic retention and the appearance of a premature stop codon. Testing of the aberrant RNA splicing was not possible due to the unavailability of the patient’s RNA or cells. Sanger sequencing confirmed the segregation of the potentially causative variant (Fig. 1), i.e., the variant is heterozygous in the proband’s parents (III:2 and III:3), his aunt (III:4) and absent in his uncle (III:1). Homozygosity mapping of proband 1 revealed that the *ATP9A* variant is embedded in a putative 6.83 Mb region of homozygosity (ROH) (chr20[GRCh37]: 45358223-52192534). While we did not find any likely pathogenic variants in known ID genes in proband IV:1 of family 2 (based on the Panelapp gene list for ID^20^; Supplementary Table 2), we cannot exclude those variants besides the *ATP9A* one might play a role in the patient’s phenotype. In particular, we identified homozygous variants in *CCDC88C* (NM_001080414.4: c.1126 C > T, p.Arg376Trp) and *ZNF407* (NM_017757.3: c.5497 > T, p.Pro1833Ser), two genes previously implicated in neurodevelopmental disorders but associated with phenotypes different than the one found in our proband. Bi-allelic variants in *CCDC88C* were associated with a form of congenital hydrocephalus^21–23^, while variants in *ZNF407* have been recently implicated in an AR form of ID with microcephaly, short stature, hypotonia, and ocular anomalies^24,25^.

## Discussion

Autosomal recessive ID is characterized by extensive genetic heterogeneity. Still, many patients do not receive a molecular diagnosis, suggesting that a considerable number of causative genes have not yet been identified^4,26^. We described three individuals from two consanguineous families with different homozygous splicing variants in canonical splice sites of the *ATP9A* gene. All three patients present with severe ID, motor delay, speech and fine motor impairment, and behavioral anomalies. Both affected sisters (IV:1 and IV:7) of family 1 had an attention deficit hyperactivity disorder-like phenotype combined with aggressiveness, whereas proband IV:1 from family 2 presented with autistic features, including prominent stereotypic movements, and lack of eye contact.

*ATP9A* is under constraint (intolerance to missense variants z-score = 4.15; pLI = 1; LOEUF = 0.2) according to gnomAD^27^. Its yeast homolog, *NEO1*, was shown to be an essential gene^28^, while the absence of the *C. elegans* orthologous *TAT-5* resulted in disrupted cell adhesion and morphogenesis in worms’ embryos^29^. Whereas ablation of the mouse orthologous *Atp9a* did not diminish survival, the *Atp9a*^*−/−*^ mice engineered and phenotyped by the International Mouse Phenotyping Consortium were hyperactive and showed a significant increased exploration in new environment reminiscent of the behavioral symptoms of our patients^30,31^. Depletion of *ATP9A* were lethal in human hepatoma HepG2 cells but not in other cell lines including HeLa, HEK293T, MCF-7, and THP-1, suggesting that the absence of ATP9A could be tolerated in certain tissues but not in others^12,15^. *ATP8A2*, another P4-ATPase highly expressed in the brain, has been implicated in a recessive disorder characterized by cerebellar ataxia, ID, and disequilibrium syndrome (CAMRQ, MIM 615268), or severe hypotonia, ID, and optic atrophy with or without encephalopathy^32–36^. A de novo balanced translocation leading to haploinsufficiency of this gene has been also proposed as the cause of moderate ID and hypotonia^37^.

Downregulation of *ATP9A* has been associated with a significant increase of extracellular vesicles release, in particular the exosome^15,16^. Extracellular vesicles release is an important form of intercellular communication that enables the transport of several different signaling molecules—including proteins and RNA—without the need of direct cell-to-cell contacts. It is involved in a wide range of biological processes, such as blood coagulation and immune response^38,39^. Different physiological roles in the central nervous system have been proposed for extracellular vesicles, including neurite outgrowth and neuronal survival^38,40^. Depletion of ATP9A reduces the plasma membrane expression of the glucose transporter GLUT1 and increases its level in the endosome, altering its recycling^12^. Deficiency of GLUT1 has been associated with a neurological disorder with a variable phenotype including epilepsy, movement disorders, mild to severe ID, and acquired microcephaly in some cases^41,42^. Similarly, alteration in the recycling endosomal processes by mutations in the *SLC9A6* sodium exchanger have been associated with Christianson syndrome (MIM 300243), a neurodevelopmental disorder characterized by ID, speech impairment, epilepsy, postnatal microcephaly, truncal ataxia, and hyperactivity^43,44^.

Since the original submission of this paper and the deposit of our data in medrxiv, a study describing additional *ATP9A* cases was published^45^. This latter study reports three affected individuals from two consanguineous families with homozygous loss of function variants, p.(Arg290*) and c.642 + 1 G > A; p.(Ser184Profs*16) in ATP9A, and phenotypic manifestations similar to our study. Patients are all presenting with mild or severe ID, motor and speech delay. Behavioral anomalies, including attention deficit, were also reported in all affected individuals. All patients were noted to have microcephaly, a feature observed only in individual IV:1 of family 1 but not in her sister, IV:7. They were all reported to have short stature and failure to thrive, which are not observed in our patients. In the other cohort, strabismus was reported for only another affected individual but not in his brother, while here it is observed in both affected Pakistani sisters. Combined with ours, these results strengthen the hypothesis of the causative role of *ATP9A* biallelic truncation variants in a novel neurodevelopmental syndrome.

In conclusion, we describe a novel AR neurodevelopmental disorder. In two unrelated consanguineous families, we identified variants predicted to affect the splicing of *ATP9A*. The three individuals homozygous for these putatively truncating variants presented with severe ID, motor and speech impairment, and behavioral anomalies. Consistent with a causative role of ATP9A in the patients’ phenotypes, *Atp9a−/−* mouse model showed behavioral changes.

## Methods

### Recruitment

The current study was approved by the IRBs of the Khyber Medical University, Peshawar, Pakistan, and the University Hospitals of Geneva, Switzerland (Protocol number: CER 11-036). Informed consent forms were obtained from guardians of all affected individuals who participated in this study. Informed consent was obtained for the publication of photos from the guardians of the affected individuals of family 1.

### Exome sequencing

The proband IV:1 of family 1 was subjected to exome sequencing (ES). DNA was enriched using SureSelect Human All Exon v6 capture kit (Agilent Technologies, Santa Clara, CA, USA) and sequenced on an Illumina HiSeq 4000 platform, with an average coverage of 120x at each nucleotide position. ES data were analyzed with an in-house customized pipeline^8^ that is based on published algorithms including BWA, SAMtools^46^, PICARD (http://broadinstitute.github.io/picard/) and (GATK)^47^. Initial screening for known or novel pathogenic mutations in the reported ID genes was performed. The 720 K SNP array was performed in parents (III:3 and III:4), affected (IV:1 and IV:7) and unaffected individuals (IV:3 and IV:5) of family 1to identify Runs of Homozygosity (ROH) using PLINK as described previously^48–50^. ROH and exome sequencing data were analyzed with CATCH^51^ to determine variants that were present in ROHs of patients (IV:1 and IV:7) but not in normal individuals of family 1. Subsequently, the variants were filtered manually by using the criteria described in published studies^49,50^.

The exome of IV:1 from family 2 was captured using the xGen Exome Research Panel v2 (Integrated DNA Technologies) and sequenced using the Illumina HiSeq 4000 platform according to the manufacturer’s protocols. The overall mean-depth base coverage was 153-fold and 97% of the targeted region was covered at least 20-fold. Read mapping and variant calling were performed as described^52^ using the Varapp software^53^. Homozygous and hemizygous variants with a MAF < 1% in the general population (1000genome, EVS, gnomAD) were retained and screened for variants in reported ID genes (Supplementary Table 1). Homozygosity mapping was performed with AutoMap, which uses Variant Call Format (VCF) files from WES^54^.

### Reporting summary

Further information on research design is available in the Nature Research Reporting Summary linked to this article.

## Supplementary information

Supplementary Information

Reporting Summary

## Data Availability

The data that support the findings of this study are available from the corresponding author upon request. The identified variants have been submitted to ClinVar under accession numbers SCV001911505-506.
