# Supplementary material for: Biallelic truncation variants in ATP9A are associated with a novel autosomal recessive neurodevelopmental disorder
Source: NPJ Genom Med. 2021 Nov 11;6:94. doi: 10.1038/s41525-021-00255-z (PMC8586153; doi:10.1038/s41525-021-00255-z)
Supplement: Supplementary file 1 — Supplementary Information [file 41525_2021_255_MOESM1_ESM.pdf]

Supplementary Table 1: Homozygous variants identified by WES and homozygosity mapping in family 1

Chr	Start	End	Ref	Alt	Gene name	Reported	Transcript	cDNA variation	Protein change	Genotype
chr20	49214156	49214156	A	G	FAM65C	yes	NM_080829	c.1739T>C	p.Leu580Arg	Homozygous
chr20	50139654	50139654	G	T	NFATC2	yes	NM_001136021	c.1066C>A	p.Pro376Thr	Homozygous
chr20	50287790	50287790	A	G	ATP9A	yes	NM_006045	c.1044T>C	p.Arg348=	Homozygous
chr20	50305602	50305602	C	A	ATP9A	no	NM_006045	c.799+1G>T	-	Homozygous
chr20	50408482	50408482	A	G	SALL4	yes	NM_020436	c.540T>C	p.Asn180=	Homozygous
chr20	50803459	50803459	C	T	ZFP64	yes	NM_018197	c.198G>A	p.Thr66=	Homozygous

Supplementary Table 2: Homozygous variants identified by WES in proband IV:1 of family 2

Chr	Start	Ref	Alt	Gene name	ID gene	Transcript	cDNA variant	Protein change	Genotype	PolyPhen-2	SIFT	CADD	AF GnomAD	Ht_gnomAD	Hom_gnomAD
chrX	19413307	C	T	MAP3K15	yes	NM_001001671.3	c.2086G>A	p.Glu696Lys	Hemyzygous	Possibly damaging (0,792)	Deleterious (0,01)	26,5	0.0001367	25	10 hemizygous
chr18	72775174	C	T	ZNF407	yes	NM_017757.3	c.5497>T	p.Pro1833Ser	Homozygous	Probably damaging (0,915)	Deleterious (0,04)	23,3	0,01407	39	0
chr1	228582429	C	T	TRIM11	no	NM_145214.3	c.1384G>A	p.Gly462Arg	Homozygous	Probably damaging (0,993)	Deleterious (0,045)	22,1	0,001619	4	0
chr20	50342357	C	A	ATP9A	no	NM_006045.3	c.327+1G>T	p.?	Homozygous	spliceAI score = 0,95	MaxEntScanDiff= 8.504; Nnsplice: wt=0,99; mut=/				
chr18	5960134	G	A	L3MBTL4	no	NM_173464.3	c.1663C>T	p.Leu555Phe	Homozygous	Probably damaging (0,999)	Deleterious (0)	20,4	0,01189	33	0
chr14	91792325	G	A	CCDC88C	yes	NM_001080414.4	c.1126C>T	p.Arg376Trp	Homozygous	Probably damaging (1)	Deleterious (0)	18,46	0,004086	10	0
chr20	47846781	C	T	DDX27	no	ENST00000371764.4	c.1019C>T	p.Pro340Leu	Homozygous	Probably damaging (1)	Deleterious (0)	17,69	\	0	0
chr1	228471296	G	A	OBSCN	no	NM_001271223.2	c.10117G>A	p.Ala3373Thr	Homozygous	Possibly damaging (0,493)	Tolerated (0,14)	15,98	0,0004023	1	0
chrX	15497935	G	T	PIR	no	NM_001018109.3	c.106C>A	p.Leu36Met	Hemyzygous	Probably damaging (0,995)	Deleterious (0,01)	14,79	0.0001828	34	22 hemyzygous
chr18	72234625	C	T	CNDP1	no	ENST00000358821.3	c.713G>T	p.Ala238Val	Homozygous	Probably damaging (0,95)	Deleterious (0)	14,48	0.004367	1235	10
chr19	1065305	G	A	ABCA7	no	ENST00000433129.1	c.6322>A	p.Glu2108Lys	Homozygous	Benign (0,167)	Deleterious (0,04)	14,44	0.0007551	212	2
chr1	222892705	A	G	BROX	no	NM_144695.4	c.305+4A>G	p.?	Homozygous	spliceAI score = 0	MaxEntScan diff = 0,140; Nnsplice; wt=1; mut=0,98	11,86	0,005335	15	0
chr8	27327274	G	A	CHRNA2	yes	NM_000742.4	c.294+4C>T	p.?	Homozygous	spliceAI score = 0,44	MaxEntScan diff = 2,753; Nnsplice = /	6,59	0,001065	3	0
chrX	47436888	T	C	SYN1	yes	NM_006950.3	c.787C>G	p.Thr263Ala	Hemyzygous	Benign (0,02)	Tolerated (0,4)	5,47	0.00002358	4	2 hemizygous
chr4	71347050	G	A	MUC7	no	ENST00000413702.1	c.589G>A	p.Ala197Thr	Homozygous	\	Tolerated (0,61)	5,1	0.0009188	258	4
chr18	73139497	C	G	SMIM21	no	NM_001037331.3	c.22G>C	p.Ala8Pro	Homozygous	Benign (0,412)	Deleterious (0,03)	5,05	0.0009199	259	1
chr22	25436977	C	T	KIAA1671	no	ENST00000358431.3	c.3880A>T	p.Pro1294Ser	Homozygous	Benign (0,085)	Tolerated (0,57)	4,47	0.00005554	2	1
chr14	93273090	G	A	GOLGA5	no	ENST00000163416.2	c.554T>A	p.Ser185Asn	Homozygous	Benign (0)	Tolerated (0,49)	3,85	\	0	0
chr1	13001085	T	C	PRAMEF6	no	ENST00000376189.1	c.598A>G	p.Lys200Glu	Homozygous	\	\	3,81	\	0	0

AF_GnomAD= Allele Frequency in GnomAD; Ht_gnomAD= Number of heterozygous individuals in gnomAD; Hom_gnomAD= number of homozygous individuals in gnomAD
